# Psychiatry in the Digital Age: A Blessing or a Curse?

**DOI:** 10.3390/ijerph18168302

**Published:** 2021-08-05

**Authors:** Carl B. Roth, Andreas Papassotiropoulos, Annette B. Brühl, Undine E. Lang, Christian G. Huber

**Affiliations:** 1 University Psychiatric Clinics Basel, Clinic for Adults, University of Basel, Wilhelm Klein-Strasse 27, CH-4002 Basel, Switzerland; andreas.papas@unibas.ch (A.P.); annette.bruehl@upk.ch (A.B.B.); undine.lang@upk.ch (U.E.L.); christian.huber@unibas.ch (C.G.H.); 2 Transfaculty Research Platform Molecular and Cognitive Neurosciences, University of Basel, Birmannsgasse 8, CH-4055 Basel, Switzerland; 3 Division of Molecular Neuroscience, Department of Psychology, University of Basel, Birmannsgasse 8, CH-4055 Basel, Switzerland; 4 Biozentrum, Life Sciences Training Facility, University of Basel, Klingelbergstrasse 50/70, CH-4056 Basel, Switzerland

**Keywords:** psychiatry, digitalization, telemedicine, computer-based cognitive behavioral therapy, app-based cognitive behavioral therapy, virtual reality, omics, electronic health records, machine learning, precision psychiatry

## Abstract

Social distancing and the shortage of healthcare professionals during the COVID-19 pandemic, the impact of population aging on the healthcare system, as well as the rapid pace of digital innovation are catalyzing the development and implementation of new technologies and digital services in psychiatry. Is this transformation a blessing or a curse for psychiatry? To answer this question, we conducted a literature review covering a broad range of new technologies and eHealth services, including telepsychiatry; computer-, internet-, and app-based cognitive behavioral therapy; virtual reality; digital applied games; a digital medicine system; omics; neuroimaging; machine learning; precision psychiatry; clinical decision support; electronic health records; physician charting; digital language translators; and online mental health resources for patients. We found that eHealth services provide effective, scalable, and cost-efficient options for the treatment of people with limited or no access to mental health care. This review highlights innovative technologies spearheading the way to more effective and safer treatments. We identified artificially intelligent tools that relieve physicians from routine tasks, allowing them to focus on collaborative doctor–patient relationships. The transformation of traditional clinics into digital ones is outlined, and the challenges associated with the successful deployment of digitalization in psychiatry are highlighted.

## 1. Introduction

The digital transformation of healthcare has spread throughout the industry. New technologies and eHealth services, such as internet health portals, telemedicine, electronic health records (EHR), online appointment-booking, and wearable fitness trackers, have become ubiquitous. The US and China, who are at the forefront of new digital technologies such as artificial intelligence, and who benefit from the sheer size of their markets, are paving the way [1].

Out of necessity for physical distancing and due to a shortage of healthcare professionals, the current coronavirus disease 2019 (COVID-19) pandemic has catalyzed the rapid and global adoption of telehealth solutions; that is, remote medical assessment and access to virtual medical care [2]. This transformation of healthcare is expected to continue beyond the pandemic [3].

Tremendous medical progress has been made in recent decades that has contributed to a significant increase in life expectancy. Between 2000 and 2016, the average life expectancy at birth increased globally from 66.5 to 72.0 years [4]. As we age, we become more vulnerable to diseases, as demonstrated by the Global Burden of Disease [5]. This is mirrored by the positive association between population aging and healthcare spending [6].

Dementia is a major driver of healthcare costs in older people. According to Alzheimer’s Disease International, around 47 million people worldwide were living with dementia in 2015, and this number is expected to almost double every 20 years, reaching 75 million by 2030 [7]. The total estimated worldwide cost of dementia is expected to rise from USD 818 billion in 2015 to USD 2 trillion by 2030 [7].

In the Global Burden of Disease 2013, mental illnesses accounted for 32% of years lived with disability (YLDs) worldwide and ranked first in terms of YLDs [8]. Furthermore, mental health disorders lead to a significantly higher utilization of healthcare resources and costs in patients with chronic diseases [9].

These demographic trends put enormous strain on the healthcare system, including psychiatry. Meanwhile, the medical world is facing a dramatic shortage of healthcare workers. The World Health Organization (WHO) estimated this shortage to be 17.4 million in 2013, including 2.6 million doctors [10]. This shortage also applies to psychiatry. The median number of mental health workers globally is approximately 9 per 100,000 people (from below one in low-income countries to 72 in high-income countries) [11]. Globally, psychiatrists remain a scarce resource, with a median number of approximately 1 per 100,000 (from 0.1 in low-income countries to 13 in high-income countries) [11]. According to the WHO, universal health coverage cannot be achieved without the support of eHealth since digitalization allows the delivery of scalable solutions to many people, even in remote locations [12].

The rapid development of new digital technologies in healthcare, the current COVID-19 pandemic, and considerable strain on healthcare systems worldwide are driving the digitalization of healthcare.

How does digitalization influence diagnostics, pharmacotherapy, psychotherapy, the doctor–patient relationship, and administrative tasks in psychiatry? Is digitalization a blessing or a curse for psychiatry? This review provides a comprehensive update on the digital transformation in psychiatry and its innovative influence on clinical practice.

## 2. Materials and Methods

A broad literature search was performed between 22 February and 31 July 2021, using PubMed, without any date restriction and with preference given to more recent articles. Search terms relevant to psychiatry, digitalization, and eHealth were included. Articles written in languages other than English were excluded. All types of articles (e.g., studies, meta-analyses, reviews, reports, editorials, and books) were considered. The bibliographies of the included publications were reviewed to provide further information. In addition, we searched the internet for information on market trends.

Given the broad scope of technologies and services covered in this review, we did not conduct a systematic review of the literature. Thus, this narrative expert review does not include all studies and publications on digitalization in psychiatry.

## 3. Results and Discussion

Below we discuss the numerous technologies and eHealth services that might be relevant for the future of psychiatry: telepsychiatry (by phone or videoconferencing, including holographic video conference systems), cognitive behavioral therapy (CBT) delivered via computer (C-CBT, typically through CD-ROM), CBT provided via the internet on a computer or mobile device (I-CBT), app-based CBT (applications downloaded from an app store onto a smartphone or tablet), virtual reality (including AVATAR therapy), digital applied games, a digital medicine system, omics, neuroimaging, machine learning, precision psychiatry, clinical decision support, EHR, physician charting (including voice dictation and digital artificially intelligent assistants), digital real-time language translators, and online mental health resources for patients (online mental health information and online physician rating).

### 3.1. Mental Health Care

#### 3.1.1. Telepsychiatry

Telepsychiatry by phone or videoconferencing can help overcome barriers to treatment, such as living in a remote or underserved area, lack of transportation, occupational or social constraints, psychological impairment, physical disability, concerns due to the potential stigma of walking into the office of a psychologist or psychiatrist, and financial difficulties [13,14,15].

There is clear evidence for the effectiveness of telepsychiatry in treating various psychiatric conditions, mainly depressive and anxiety disorders, using cognitive and behavioral therapy, as well as for enhancing medication adherence [16]. Furthermore, telepsychiatry has been shown to allow volume-sensitive cost-saving [16].

Several studies have reported that clinical effectiveness, treatment adherence, and patient satisfaction were comparable between telepsychiatry and face-to-face therapy [14,17,18,19,20,21]. Thus, telepsychiatry represents a viable option for providing treatment to people with limited or no access to mental healthcare resources [22].

In the Accenture 2020 Digital Health Consumer Survey conducted before the COVID-19 pandemic, 19% of US consumers had already received treatment through telemedicine from traditional healthcare providers, while 54% were willing to receive virtual medical care [23]. Thus, the use of telemedicine among US consumers was quite uncommon before the COVID-19 pandemic, despite rapid uptake since the beginning of the millennium [24]. Telemedicine has been boosted by the pandemic, since virtual healthcare allows the avoidance of face-to-face clinic visits, hence reducing the risk of exposure to the COVID-19 virus [23,25,26].

The psychological burden of the pandemic has also driven the use of telepsychiatry in various groups of people, such as those in isolation, caring families and friends, those facing the death of loved ones, people in quarantine, those confined in constrained spaces, those suffering from social isolation, and people with financial difficulties or job losses [27]. People with psychiatric disorders represent a high-risk population because they are more vulnerable to stress than the general population [28].

In the USA, mental health care delivered via telepsychiatry increased from less than 1% of visits (in-person and telepsychiatry) before the pandemic to 56% in December 2020 [29,30,31]. During the transition from face-to-face therapy to telepsychiatry, the volume of outpatient encounters initially decreased before rebounding to pre-pandemic levels [32]. However, the digital divide led to disparities in access to telepsychiatry (particularly video visits)—for instance, owing to a lack of broadband internet availability, inability to afford suitable devices, lack of digital literacy, cognitive deficits, or mistrust of technology [32,33,34].

In a systematic review of the literature on telemedicine, Ekeland et al. reported limited evidence for the effectiveness of telemedicine in patients with chronic conditions [35]. They recommended that larger and more rigorous controlled studies be conducted, including the standardization of methodological aspects [36]. The Model for Assessment of Telemedicine (MAST) represents such a framework [37]. Its development followed an initiative by the European Commission, which supports the design of guidelines for consistent assessment of the outcomes of telemedicine [37,38,39].

A new, innovative technology is about to change telepsychiatry. In May 2021, Google presented a holographic video communication system that allows users to see a 3D hologram of the person they are talking to [40]. Google describes the new technology in the following way: “Imagine looking through a sort of magic window, and through that window, you see another person, life-size, and in three dimensions. You can talk naturally, gesture, and make eye contact” [40]. Interestingly, Google indicated that they tested the new technology with healthcare companies [40]. Its impact on telepsychiatry could be tremendous.

It remains unclear how much each communication channel contributes to a message’s impact, including verbal, vocal, facial, and gestural channels [41]. In any case, nonverbal cues play a critical role in message interpretation, such as when a verbal message is ambiguous [42]. However, as Schmid Mast emphasized, one should remain cautious about interpreting nonverbal cues, since one and the same nonverbal behavior can mean different things depending on the context [42].

Nonverbal communication is crucial to the doctor–patient relationship [42]. On the one hand, the patient’s nonverbal behavior is critical when diagnosing psychiatric disorders [43]. On the other hand, the physician’s nonverbal behavior also has an impact on patients. For instance, Schmid Mast pointed out that nonverbal behavior indicative of a patient-centered approach (e.g., more gazing, more forward leaning, more nodding, more gestures, and non-dominant tone of voice) was related to patient satisfaction [42]. DiMatteo et al. reported that physicians with strong nonverbal communication skills had patients who were more compliant to scheduled appointments [44].

#### 3.1.2. Computer-Delivered and Internet-Based Cognitive Behavioral Therapy

Cognitive behavioral therapy (CBT) delivered via computer (C-CBT, typically through CD-ROM) and CBT provided via the internet on a computer or mobile device (I-CBT) represent an avenue that shows the potential of digitalization in psychiatry. For instance, they can be used to treat depressive disorders, bipolar disorders, generalized anxiety disorders, panic disorders, social anxiety disorders, phobias, obsessive–compulsive disorders, post-traumatic stress disorders, and adjustment disorders [45].

The use of I-CBT is driven by the increasing access to the internet and mobile phones worldwide. In 2019, 51% of the world’s population was using the internet [46]. In the same year, there were approximately 105 mobile-cellular subscriptions per 100 inhabitants worldwide [46]. These figures show that instant access to the internet through smartphones represents a therapeutic opportunity to address psychological symptoms and distress as they arise in daily life [15].

C-CBT and I-CBT include different kinds of interventions. Patients can receive psychoeducation, analyze risk situations and triggers, capture and rate their thoughts and emotions, record their activities, and learn specific skills, such as coping, problem-solving, social skills, and cognitive restructuring. They can keep a sleep diary; perform exercises, such as exposure, relaxation, or mindfulness meditation; receive automated text messages or emails; and participate in online discussion forums [15,47,48]. Some therapies are designed to be used on a standalone basis, while others can be combined with face-to-face therapy to varying degrees [15]. For example, I-CBT can be supported by a therapist who spends a limited amount of time guiding the patient through the modules (guided I-CBT).

There is moderate to strong evidence for the effectiveness of C-CBT and I-CBT on measures of depression and anxiety [21,49,50]. The NICE Guidelines for depression recommend guided C-CBT and I-CBT for people with persistent subthreshold depressive symptoms or mild to moderate depression [51].

The effect sizes of C-CBT and I-CBT, compared with conventional face-to-face CBT, were shown to be equivalent [21,47,52,53,54,55]. Andrews et al. reported that face-to-face CBT required 7.8 times more therapist time than I-CBT [53].

There is increasing evidence for the efficacy of C-CBT and I-CBT for alcohol-use disorders [48,56]. Conversely, evidence for the effectiveness of C-CBT and I-CBT in people with schizophrenia (e.g., for psychoeducation, monitoring early warning signs of relapse, enhancing medication adherence, improving socialization, and reducing auditory hallucinations) remains limited [57,58,59].

#### 3.1.3. App-Based Cognitive Behavioral Therapy

More than 200 healthcare applications (apps) for smartphones and tablets are uploaded daily to Apple’s App Store and Google Play [60] (p. 93). Approximately 300,000 health apps are currently available, of which 10,000 to 20,000 are mental health apps [61,62]. These publicly available apps are opening further opportunities to provide mobile healthcare (mHealth) anytime, anywhere. As for C-CBT and I-CBT, some mental health apps are designed to be used on a standalone basis, while others can be combined with face-to-face therapy to varying degrees [15]. According to Huckvale et al., app-based interventions are likely to be much more impactful if used in conjunction with face-to-face therapy, rather than being implemented in isolation [63].

App-delivered interventions have been shown to be acceptable in treating people with mental health problems [64,65]. Their efficacy has been demonstrated in the treatment of depression and anxiety [66,67]. Strong evidence is available for the treatment of substance abuse disorders and chronic insomnia (see below) whereas evidence with patients with schizophrenia and post-traumatic stress disorder remains unclear [68,69,70].

Smartphone apps for suicide prevention have the potential to contribute to the reduction of suicide attempts and deaths through different kinds of interventions, such as screening for suicide risk, developing coping skills and emotional regulation strategies, providing emergency contact details, facilitating access to psychotherapy, encouraging people at risk to obtain support from family and friends, or developing a safety plan [71,72]. App-delivered interventions seem to be effective in reducing suicidal ideation immediately following the intervention phase [73]. However, evidence of the effectiveness of smartphone apps in reducing suicide plans and attempts remains unclear [72,73]. Torok et al. recommended that digital interventions for suicide prevention be widely promoted because of their potential impact if uptake is widespread [73].

Just-in-time adaptive interventions (JITAI) go one step further by integrating real-time data [63,74,75]. The latter include self-reports (so-called “active data”, e.g., regarding mood regulation, sleep, hallucinations, medication adherence, substance use, and social functioning) and smartphone sensor data (so-called “passive data”, e.g., GPS, actigraphy, fitness tracker, patterns of typing and scrolling, or voice and speech analysis) to infer the patient’s context and “digital phenotype” (cognition, emotions, and behavior). The app then responds with customized interventions, such as notifications, encouraging physical activity, alerts asking the patient to walk away from his or her usual liquor store, or messages recommending that the patient take their medication. In the coming years, the Internet of Things will lead to an increasing number of sensors, cameras, and microphones in cars, homes, and everyday objects capable of transmitting real-time passive data [76].

Potential uses of JITAI are manifold. For instance, Iliescu et al. suggested that JITAI could be used to bridge the gap between discharge from an inpatient psychiatric unit and the following outpatient care, as this has been shown to be a high-risk period [77].

Smartphone apps still have a few hurdles to overcome before they can be widely integrated into clinical practice. Tonning et al. pointed to methodological challenges in randomized controlled trials on smartphone-based treatment in psychiatry, showing that the trial design and reporting were lower in quality compared with classic medical randomized controlled trials [78].

Furthermore, Larsen et al. showed that there is a lack of evidence from app-specific studies, and many mental health apps publicly available in app stores describe techniques for which there is no clear evidence in the literature [79]. This overall lack of evidence was confirmed by Lagan et al. in a recent review of 278 publicly available mental health apps [80].

The use of some mental health apps may be associated with safety issues. Thus, Larsen et al. identified apps for suicide prevention with potentially harmful content (e.g., list of means of instant death, although those means were presented as suggestions for removing access to such means) [71]. Martinengo et al. assessed the adherence of advice on suicide prevention in depression management and suicide prevention apps to evidence-based guidelines from the UK, USA, and WHO [81]. They found that only 7% of the apps incorporated all six recommended suicide prevention strategies (psychoeducation, tracking of mood and suicidal thoughts, offering activities to deter suicidal thoughts, safety plan development, access to support network, and in-app access to emergency counseling). The remaining apps were potentially inadequate for managing people at risk of suicide. Two apps available in Google Play and Apple’s App Store and downloaded more than one million times each provided erroneous crisis helpline numbers.

According to Sucala et al., there is a significant need to develop guidelines for apps marketed for mental health [82]. This need has led to the development of app-rating guidelines, such as the Mobile App Rating Scale (MARS) and the American Psychiatric Association’s app evaluation framework [83,84]. Unfortunately, the increasing number of app evaluation frameworks makes it difficult for clinicians and patients to select an appropriate evaluation framework and find an appropriate mental health app [62]. Therefore, app-rating platforms, such as PsyberGuide, have gone one step further by identifying, describing, and rating publicly available mental health apps [85].

Singh et al. suggested that medical professional societies could inform patients through labels [86]. The former could provide information to the latter about the apps and the conditions they target, a description of their functionalities, and warnings in case of safety or privacy issues [86]. In the UK, the NHS provides an app library, with a section dedicated to mental health [87].

In 2017 and 2018, the technology company Pear Therapeutics reached a milestone in the development of evidence-based health apps by receiving authorization from the US Food and Drug Administration (FDA) for two apps providing CBT for substance-use disorders [88,89,90]. In 2020, the same company received market authorization from the FDA for an app providing CBT for chronic insomnia [91]. The clearance was supported by two randomized controlled trials [92,93]. Through these clearances by the FDA, new quality standards have been established in the health app industry.

The privacy of personal digital data is critical to the uptake of smartphone mental health apps [94]. Complex privacy policies, lack of transparency regarding data sharing, inadequate efforts to secure users’ consent, and the ability of some companies to aggregate highly diverse data and uniquely identify and profile users, remains a concern [95,96,97]. The risk of deductive disclosure grows as an increasing number of columns of de-identified big data from different domains (e.g., EHR, wearable devices, administrative data, GPS, search term records, social media posts, or cell phone records) are merged, making individuals re-identifiable by means of artificial intelligence [98]. Several authors have pointed to the role of regulators in addressing these privacy issues [15,96,97].

In the Accenture 2020 Digital Health Consumer Survey, the authors reported that the use of health apps among adults in the USA dropped from 48% to 35%, while the use of wearable devices, such as fitness trackers, fell from 33% to 18% in 2018 and 2020, respectively [23]. Accenture pointed to concerns about privacy and data security as a key barrier to the adoption of digital health technologies. Thirty-five percent of the respondents were not confident that their digital healthcare data were used responsibly and in their best interests, while 55% did not trust technology companies to keep their digital information secure. McKee et al. identified cyberattacks as a potential threat to data security in relation to the digitalization of healthcare [99].

Poor user-friendliness of some apps represents another barrier to the uptake of mental health apps [100,101]. In the Accenture 2020 Digital Health Consumer Survey, 50% of the surveyed US healthcare consumers indicated that a cumbersome digital experience with a healthcare provider ruined the entire experience with that provider [23]. In this context, Wilhelm et al. emphasized the need for the involvement of key stakeholders in the development of mental health apps, such as patients, clinicians, designers, engineers, and representatives from payers [15].

Torous and Vaidyam suggested an innovative way to solve challenges around evidence-based recommendations of health apps, safety, privacy, user-friendliness, and compatibility with older or cheaper phones [102]. They suggested a collaborative approach aimed at developing a single, open-source app that provides the core functions users expect from many digital health apps—an app with multiple uses instead of multiple apps [102]. This led to the mindLAMP project [103].

The integration of digital healthcare and traditional medical services into “digital clinics” may be critical to the uptake of health apps [102]. In the Accenture 2020 Digital Health Consumer Survey, 54% of US consumers were willing to receive virtual care from traditional medical care providers, versus 27% for virtual services from technology or social media companies [23].

A further challenge faced by digital CBT is adherence [15]. This is similar to the difficulties related to some patients’ engagement in face-to-face psychotherapy or medication adherence. Involving mental health clinicians into the app-delivered treatment process is critical for enhancing engagement [15]. Peer support platforms integrated with digital CBT therapies may also prove to be helpful in improving engagement [15]. Fitzpatrick et al. demonstrated that the use of a chatbot may represent an opportunity to enhance adherence in I-CBT and app-delivered interventions by mirroring the therapeutic process and experience through a fully automated conversational agent [104]. The latter delivered CBT through brief daily conversations and mood tracking. The bot also provided the user with weekly charts that described the user’s mood over time and was supported by a vocal comment.

#### 3.1.4. Virtual Reality

Virtual reality (VR) creates a digital environment that replaces a user’s real-world environment [105]. VR relies on increasingly sophisticated systems that use equipment such as computers, smartphone apps, headsets, motion chairs, gloves, and sensors [106]. VR has had a significant impact on many industries outside of gaming and entertainment, including healthcare [105]. It is only the first step towards digital reality, which includes the following: VR (the digital environment replaces the user’s real-world environment); augmented reality (digitally created content is built into the user’s real-world environment); mixed reality (digitally created content is integrated into the user’s real-world environment where both coexist and interact); immersive reality (multisensory digital experience); and 360° video (the user can look in every direction) [105].

VR immerses the patient in a digital virtual environment and exposes them to a specific fear stimulus. There is clear evidence for the effectiveness of VR-based interventions in treating agoraphobia, specific phobias (e.g., acrophobia, arachnophobia, and aviophobia), and social anxiety disorder [107,108,109,110]. The effect sizes are comparable to those of traditional CBT [107,108,110].

However, there is limited evidence demonstrating the effectiveness of VR-based exposure therapy in treating posttraumatic stress disorder [108,111]. The effect size seems to be comparable with that of standard evidence-based interventions [108,111].

Leff et al. developed AVATAR therapy for patients with psychotic conditions, such as schizophrenia, who experience refractory auditory verbal hallucinations [112]. Craig, one of the pioneers of AVATAR therapy, describes the novel treatment as follows [113]: The therapy is based on a three-way conversation between therapist, patient, and a digital simulation or avatar (including visual representation and voice) of the hallucinated voices. The software changes the therapist’s voice into a close match of the hallucinated voice. In a randomized controlled trial of AVATAR therapy, Craig et al. found a large effect size in reducing the severity of persistent auditory verbal hallucinations [114]. Ward et al. suggested that transdiagnostic interventions should be considered [115]. Based on a Cochrane review by Aali et al., evidence remains unclear and large, sufficiently long-term, and well-designed randomized trials are still needed [116]. While the original AVATAR therapy has been using 2D virtual avatars, the use of 3D avatars is now being studied [117].

Preliminary findings suggest that further VR-based interventions may prove useful in patients with psychosis—for example, the opportunity for the physician to help patients observe their cognitions, emotions, and behaviors in a controlled environment and modify them [118].

However, despite these advances in VR technologies, users can experience VR sickness, in particular, disorientation, oculomotor symptoms, and nausea [119].

#### 3.1.5. Digital Applied Games

VR is driving gamification (i.e., enhancement of the service through gaming elements) and the use of digital applied game interventions (i.e., “serious” games) in psychotherapy [120]. The gamification of psychotherapy and the use of applied games benefit from the halo effect of the video game industry. According to the Entertainment Software Association, 64% of adult Americans are video game players and 75% of households have at least one video game player [121]. According to the same report, video game players believe that video games have a positive impact on their lives. For instance, 79% reported that games provide relaxation and stress relief. Smartphones represent the most common device used for video game play among adult players [121]. This makes access to applied game interventions instant and global.

Gamification elements in apps include, for instance, levels or progress feedback, points or scoring, rewards or prizes, narratives or themes, and personalization [122]. Gamified apps most commonly target substance use, depression, and anxiety disorders [122]. They usually aim to increase engagement with an intervention and enhance its intended effects [122].

Given the appeal of video games in the general population, applied games may help address some of the challenges faced by mental healthcare [120]. They may contribute to enhancing engagement and hence adherence to psychotherapy through the addition of gaming elements; to the global accessibility of psychotherapy through the use of smartphones; to the availability of psychotherapy despite the global shortage of mental health specialists; and to circumventing the stigmatization of psychiatry and psychotherapy through preserving anonymity [120].

At the same time, one should beware of issues such as data security and privacy, as well as misuse and abuse of applied games with people who are already inclined towards unhealthy use of technology [120]. Furthermore, there is limited evidence supporting the benefits of using applied games in mental healthcare [120,123]. A major challenge is the transfer of skills developed through applied games to real-world situations [124].

#### 3.1.6. Digital Medicine System

According to the WHO, adherence can be defined as the extent to which a person’s behavior—taking medication, following a diet, and/or executing lifestyle changes—corresponds with agreed recommendations from a healthcare provider [125]. Medication non-adherence is a major issue in psychiatry. A 35% rate of non-adherence was reported for antipsychotics, along with 46% for antidepressants, 35% for sedative-hypnotics, 38% for anxiolytics, and 45% for mood stabilizers [126]. Geretsegger et al. reported that only 25% of psychiatric patients admitted to the hospital had plasma levels of psychotropic medications in the expected range [127].

Forgetting to take medication seems to be the main reason for non-adherence [126]. Several risk factors for non-adherence in patients with mental disorders have been identified, such as younger age (<40 years), marital status (unmarried or living alone), weaker social support, lack of insight into own illness, severity of symptoms, cognitive deficits, substance use, negative beliefs about the medication, influential beliefs of others, side effects, treatment complexity, medication cost, poor access to medical care, and poor doctor–patient relationship [128,129].

Non-adherence compromises treatment, jeopardizes the patient’s safety, and increases healthcare costs [125]. Thus, non-adherence to antipsychotic medication has been shown to be associated with a higher rate of psychiatric and medical hospitalizations in patients with schizophrenia [130]. Biochemically verified non-adherence to antipsychotic medication was reported to be associated with an increased risk of completed suicide [131]. In the USA in 2016, the estimated cost of drug-related morbidity and mortality resulting from non-optimized pharmacotherapy, including medication non-adherence, was USD 528 billion [132].

Various strategies can be used to enhance medication adherence, such as patient education, support from family and friends, improved doctor–patient relationships, employment, simpler drug regimens (e.g., drugs with longer half-lives, extended-release formulations, long-acting injectable medications), and contingency management (i.e., reinforcement of adherence through incentives) [128,133,134,135].

In this context, the use of apps to support medication adherence has attracted much interest. These applications are based on various features, such as patient education, medication reminders, documentation, feedback messages, data statistics, and appointment reminders [136]. In a systematic review and meta-analysis, Armitage et al. reported that medication adherence interventions delivered by smartphone applications were associated with higher self-reported medication adherence (OR 2.120, 95% CI 1.635–2.747) [137]. However, this result should be considered with caution due to methodological limitations [137]. Peng et al. also reported a significant improvement in medication adherence through mobile applications (Cohen’s d = 0.40, 95% CI 0.27–0.52) although they pointed out the general dearth of evidence.

The digital medicine system (DMS) represents a novel and quite radical step towards further improvement of medication adherence. The DMS includes the following elements [138,139,140,141,142,143]: First, a sensor smaller than a sesame seed, contained in the pill and activated upon interaction with stomach fluid, is ingested. After activation, the sensor sends a signal with a specific code. The sensor is made of ingredients found in the food supply and is eliminated through feces. Second, an adhesive patch placed on the patient’s skin detects the signal transmitted by the sensor. The patch also monitors various health information, such as heart rate and number of steps; that is, physical activity. Third, a mobile device application collects data from the patch and sends them to the cloud. Patients can access these data through the mobile application, while physicians can view them on an internet portal. The application also reminds patients to take their medication as prescribed.

In psychiatry, the DMS has been investigated in three small studies in patients with schizophrenia, bipolar disorder, and major depressive disorder [138,139,141,143]. In one of those studies, the ingestible sensor was embedded within tablets containing a placebo [138]. In the other two studies, the sensor was embedded within pills containing various dosages of aripiprazole [139,141]. The reported medication adherence ranged from 74% to 89% [138,139,141,143]. The DMS was generally well-tolerated. A high proportion of patients (78–89%) and healthcare providers (72%) were satisfied with the DMS [138,139].

According to Cosgrove et al., approval by the FDA in 2017 of a version of aripiprazole embedded with an ingestible sensor was based on weak evidence [144,145]. There was no prospective, double-blind, randomized controlled trial comparing digital aripiprazole with a non-digital formulation or placebo [145]. In addition, controlled trials are needed to investigate treatment outcomes, such as remission rate and quality of life, to understand the clinical impact of digital aripiprazole [143]. Furthermore, digital aripiprazole needs to be tested in severely ill patients, since published studies enrolled patients who were moderately ill and in stable condition on non-digital oral aripiprazole [143].

### 3.2. The Promise of Big Data

#### 3.2.1. Omics

Numerous biomarkers are used in psychiatric clinical practice, for example, the 42 amino acid form of amyloid β (Aβ42), total tau (T-tau), phosphorylated tau (P-tau), and the neurofilament light protein (NFL) in the cerebrospinal fluid of patients with Alzheimer’s disease [146].

Biomarkers from the field of omics (e.g., genomics, epigenomics, transcriptomics, proteomics, and metabolomics) are expected to play an increasing role in psychiatry. The term “omics” refers to the study of the roles and relationships of various types of biological molecules [147]. Omics will hopefully pave the way to precision psychiatry, for example, by understanding the mechanisms contributing to the development of psychiatric disorders, by predicting the risk of developing specific psychiatric disorders, through the early detection of psychiatric disorders, and through personalized pharmacotherapy.

Omics generate big data, whose processing has been enabled through digitalization and a quantum leap in computational sciences. The size of the genome sequence (approximately 3 billion nucleotides distributed over 23 pairs of chromosomes) illustrates the huge amount of data that needs to be collected and analyzed [148].

Most psychiatric disorders are at least moderately heritable [149]. Heritability is a population-derived value. It estimates the proportion of average individual variation in a trait that is explained by inherited factors, while the rest of the variation is explained by non-inherited factors and measurement error [150]. For instance, the heritability of bipolar disorder reaches up to 87% [151]. Heritability of schizophrenia has been reported to be approximately 80% [152]. A heritability of 58–79% was found for Alzheimer’s disease [153]. Inherited factors contribute as much as 67% to the development of major depressive disorder [154]. Twin, family, and adoption studies have shown that the heritability of alcohol dependence amounts to 50–60% [155]. Heritability of borderline personality disorder is estimated to be 46% [156]. Panic and generalized anxiety disorders have lower rates of 43% and 32%, respectively [157].

Genomics focuses on the study of genes, respectively on the variation in DNA sequences. Although new technologies have allowed the deciphering of the genetic code of humans and identification of numerous genetic variants associated with various medical conditions (e.g., in oncology), identification of disease-specific gene mutations and further underlying DNA alterations remains difficult [147]. Most psychiatric disorders are associated with potentially thousands of genetic variants, each contributing a small effect (OR mostly between 1.01 and 1.2, with some rare exceptions) [158] (p. 55). Further, up to 45% of psychiatric patients have more than one psychiatric disorder [159]. Comorbidity is assumed to be partially due to pleiotropy (i.e., a single gene or variant being associated with more than one psychopathological trait) or a polygenic predisposition to psychopathology [149,160]. These challenges have paved the way for other omics approaches.

Epigenomics studies chemical modifications of the genetic sequence. Epigenetics encompasses an ever-growing set of potentially heritable changes in the genome that can be induced by environmental events and regulate gene expression without changing the underlying DNA sequence [161,162]. Such environmental factors include, for example, poor nutrition, reduced food availability, toxic chemicals, psychosocial stress, and physical stress [161,163]. Epigenetic mechanisms include, for example, DNA methylation, histone modifications, and non-coding RNAs [163]. Epigenetic modifications can occur in both somatic and germ cells. In the latter, gametes may carry the DNA sequence and epigenetic modifications from parents to offspring [163]. Epigenetic changes can also occur at an early developmental stage in the embryo [163]. As in the case of genetic mutations, epigenetic modifications might have a negative impact on the individual [163]. To reduce the negative consequences of epigenetic changes in offspring, most epigenetic alterations are corrected through reprogramming during gametogenesis or immediately after fertilization [163].

Transcriptomics—the analysis of cellular RNA transcripts—allows the determination of how changes in gene transcription correlate with the onset or progression of diseases [147]. However, risk loci on genes that have been associated with psychiatric disorders are located in both coding and noncoding portions of the genes [158] (pp. 7–13). Therefore, transcriptomics does not allow us to shed light on all variants of DNA sequence that are associated with the development of psychiatric disorders.

While transcriptomics focuses on the immediate product of gene transcription, proteomics analyzes cellular proteins; that is, the ultimate product of gene expression. Such proteins include, for example, receptors, ion channels, transporters, and metabolizing enzymes in the dopaminergic, adrenergic, serotonergic, glutamatergic, GABAergic, cannabinoid, and opioid systems, as well as neurotrophins and peptide hormones [158] (p. 55). However, studies have shown a relatively low correlation between protein expression and RNA transcripts [147]. Protein synthesis seems to be subject to modulation through numerous mechanisms that make the use of proteins as disease-specific biomarkers difficult.

Metabolomics analyzes small intracellular molecules whose synthesis is mediated by proteins. The difficulty in identifying disease-specific biomarkers also applies to metabolomics [147].

Thus, each omics approach taken separately faces considerable challenges with the characterization of disease-specific biomarkers. Therefore, multi-omics approaches that look for correlations across large sets of multiple omics data from the same patients in very large study populations (tens of thousands of participants) may advance precision medicine [147]. Therefore, precision psychiatry, including pharmacotherapy, might benefit from the digitalization of healthcare and the ability of computational sciences to process massive amounts of data.

#### 3.2.2. Neuroimaging

Neuroimaging is another promising biomarker source. For instance, low striatal dopamine transporter uptake on single-photon emission computed tomography (SPECT) or positron emission tomography (PET) scans can be helpful in diagnosing dementia due to Lewy body disease [158] (pp. 162–163). However, the translation of neuroimaging biomarkers into psychiatric clinical practice remains limited mainly to the diagnosis of neurocognitive disorders [158] (p. 161). Otherwise, the role of neuroimaging in psychiatric clinical practice is limited to excluding structural lesions such as a tumor, stroke, cerebral hemorrhage, or brain malformations [164]. In the future, digitalization will foster multisite big-data approaches, machine learning, and the association of neuroimaging phenotypes with other biomarkers, such as omics data [164,165,166,167,168]. This may help in identifying neuroimaging biomarkers that can be used in psychiatric clinical practice.

#### 3.2.3. Machine Learning

##### Definition of Artificial Intelligence and Machine Learning

Artificial intelligence (AI) can be defined as the simulation of human intelligence in machines, such as computers and robots, which are programmed to mimic human cognitive functions such as learning and problem-solving [169]. Machine learning (ML) is a subset of AI that uses algorithms that learn from training data before predicting outcomes for new real-world data [169,170].

Access to big data from multiple sources (e.g., EHR, genomics, wearable devices) combined with increasing computing power enable the emergence of deep learning models. Humans impose fewer assumptions on the algorithm, moving away from carefully designed statistical models to allow the computer to identify subtle and complex patterns that are unavailable with traditional analytic approaches and create increasingly accurate models directly from raw data [170,171].

As Beam and Kohane put it, ML is a natural extension of traditional statistical approaches [171]. While conventional statistical methods sequentially assess single predictor variables, ML can integrate multiple variables and assess patterns of interactions among these variables [172]. Besides, the focus of ML is different from that of classical statistical methods. ML focuses on prediction, while traditional statistical methods focus on the variance of group effects (e.g., healthy subjects versus psychiatric patients, placebo treatment versus new treatment) [173]. Finally, deep learning models can generate new hypotheses by identifying novel associations, while traditional statistics simply confirm or reject the null hypothesis [174]. Deep learning is also used in the world of tech giants, such as Google, Facebook, and Apple.

Another potential source of data for ML may come from natural language processing (NLP), another subset of AI, which allows the transformation of unstructured clinical text, such as clinical notes from EHR and conversations with patients (using speech recognition), into structured clinical data [170,175].

As Stead advised, the clinician should view the output of ML as a statistical prediction that may be wrong, judge whether the prediction applies to their patient, and decide if additional data or expertise is required to make an informed decision [176].

ML has been investigated in various psychiatric disorders. This review presents a few examples in detail.

##### ML for the Assessment of Suicide Risk

According to the WHO, approximately 800,000 people die by suicide every year worldwide; that is, approximately one person every 40 s [177]. A vast majority of people who commit suicide have a mental disorder (87% according to a study by Arsenault-Lapierre et al.), mainly depression, followed by disorders due to substance use, schizophrenia, and personality disorders [178,179]. The rate of suicide among psychiatric in-patients is relatively low. A study found a rate of 13.7 per 10,000 admissions; that is, below 0.2% [180]. Conversely, the risk of suicide is markedly increased after discharge from the hospital, particularly in the immediate post-discharge period. Chung et al. reported a post-discharge suicide rate of 484 per 100,000 person-years [181]. The suicide rate was highest within 3 months after discharge (1132 per 100,000 person-years) and remained high for many years.

A significant proportion of people who commit suicide have contact with healthcare providers before their suicide. Luoma et al. reported that the rate of contact with mental health services was 19% within the month before suicide and 32% within a year before suicide [182]. The rate of contact with primary care providers was 45% in the month before suicide and 77% within a year of suicide. Unfortunately, patients often conceal or deny suicidal thoughts before attempting suicide, or are unable to accurately assess their emotional states or future risk of suicide [183]. In a study by Isometsä et al., only 22% of the people who had seen a healthcare professional within a month before suicide had communicated their suicidal ideations or intent during the last appointment [184].

The limited predictive value of patients’ self-reports has fueled decades of research on predictors of increased suicide risk (e.g., history of self-harm, family history of suicide, or being male) [185]. Although numerous risk factors for suicide have been identified, the usefulness of suicide risk assessment scales in clinical practice remains limited. Risk assessment scales lack sufficient sensitivity and specificity to be clinically reliable [186,187,188]. Their predictive accuracy is similar to clinical risk assessment and is insufficient for use in clinical practice [189]. They are even potentially harmful, as they provide false reassurance to clinicians [186,190]. Furthermore, risk assessment instruments do not allow us to predict when someone will attempt suicide. The time between the first current suicidal thought and the accomplishment of a suicide attempt is usually short. Deisenhammer et al. reported that this period lasted 10 min or less in nearly half of suicide attempters [191].

Given the lack of accuracy of clinical risk assessment and risk assessment instruments for suicidality, numerous studies have investigated the use of ML in detecting suicide risk. They have used various sources of data, for example, EHR, medical discharge notes, NLP (key words and acoustic features), and data from social media [192].

According to Bernert et al., ML seems to reach a high level of accuracy (sum of true positive and true negative assessments, divided by the number of all assessments) in the prediction of suicidal behavior (>80%) [192]. In a meta-analysis of 87 studies, risk assessment through ML outperformed risk stratification based on clinical assessments. However, the positive predictive value (PPV) (number of correctly predicted positive cases divided by the number of predicted positive cases) of prediction models for suicide attempts and deaths remains extremely low. In a systematic review of 17 studies, Belsher et al. found a PPV of ≤1% for suicide mortality despite good accuracy (≥80%) [193]. In other words, ML algorithms still deliver a high rate of false alarms despite a high level of accuracy.

In that respect, the impact of the low prevalence of suicide deaths on the computation of accuracy and PPV should be emphasized. When the prevalence of an outcome is low, high accuracy can be achieved despite low sensitivity through the high specificity of the predictive model [194]. Such a pattern was reported by Barak-Corren et al. in a large retrospective study investigating whether longitudinal data from EHR can be useful for predicting the future risk of suicidal behavior (90–94% accuracy and 33–45% sensitivity at 90–95% specificity) [195]. Therefore, it is generally accepted that accuracy is not the metric of first choice when describing model performance [194]. The model performance should rather be reported in terms of sensitivity, specificity, and area under the receiver operating characteristic curve (AUC) [194].

As demonstrated by Cox et al., PPV changes as a function of prevalence, with lower prevalence being associated with lower PPV [194]. This applies to suicide deaths with a global age-standardized suicide rate of 10.5 per 100,000 population in 2016 [177]. The negative impact of low prevalence on PPV is likely to be even stronger if prediction models are used to identify individuals at risk of short-term suicide (e.g., to avoid unnecessary restrictive care such as involuntary hospitalization) due to even lower base rates of short-term suicide attempts and deaths [196].

Nevertheless, ML has the potential to improve the assessment of suicide risk—for example, by identifying new risk factors, the dynamics of risk factors, and complex patterns of interacting risk factors [196,197]. However, these tools need to be tested in actual care settings, including health outcomes, healthcare costs, and adverse outcomes [193]. In this context, psychiatrists, who interpret the results of predictive models and those who build predictive models for suicide, need to work hand in hand and have a working understanding of ML in assessing suicide risk [194].

More accurate suicide risk assessment through ML may lead to improved suicide prevention through timely psychotherapy and targeted pharmacological interventions, such as lithium for patients with bipolar or depressive disorders, esketamine for patients with depressive disorders and active suicide ideation, and clozapine for patients with schizophrenia [183,198,199,200,201]. Psychotherapy and pharmacological interventions should be deployed on the basis of a strong therapeutic alliance [183,202,203]. Limited supportive contact through brief caring digital text messaging does not suffice [204]. The therapeutic relationship remains fundamentally analog.

The contribution of ML to suicide prevention may be even stronger if it is applied beyond the boundaries of the healthcare system. According to Pompili et al., nearly half of the people who commit suicide communicate their suicidal intentions to healthcare professionals or next of kin prior to their suicide, either through verbal, written, or behavioral communication [205]. According to the authors, this proportion is likely to be underestimated. Teenagers and young adults often share their suicidal thoughts with the public on social media platforms such as Facebook and Twitter [206]. Thus, ML can be used to predict suicide risk based on information posted on social networks [207]. This has led a few platforms such as Facebook and Twitter to set up teams that contact people whose posts are overtly suicidal and provide them with support and resources [206]. Additionally, healthcare professionals can view a patient’s social media posts to assess suicidality more accurately. However, this approach is associated with ethical and privacy issues that need to be considered [206].

In the future, big omics data may help identify people at risk of suicide and develop new treatments. Heritability of suicide attempts has been shown to be approximately 4% [208]. Furthermore, a higher polygenic risk score for depression has been demonstrated to be associated with an increased risk of suicide attempts across psychiatric disorders [209]. Owing to increasingly large sets of omics data, the number of genetic associations is expected to increase. This may ultimately translate into a better understanding of suicidality, improved prevention, and new treatments [209].

##### ML for the Prediction of Therapeutic Outcomes in Depression

Depression is a leading cause of disability-adjusted life years (DALYs) worldwide. According to the Global Burden of Disease 2019, depression ranked 13th among 369 diseases and injuries (10th without neonatal disorders, congenital birth defects, and road injuries) [5]. This represents a significant progression in comparison with the 1990 results, where depression ranked 19th. Trivedi et al. reported that patients with a clinically significant reduction in symptom severity, following treatment with an antidepressant, experienced a significant reduction in work-related disability [210]. In contrast, patients who remitted in the second treatment trial continued to have impairments at work.

In this context, there has been a growing interest in identifying predictors of therapeutic outcomes. However, the available evidence remains insufficient to support the use of any single predictor variable to guide the treatment of depression [172]. Thus, pharmacogenomics shows promising results, although currently available evidence remains limited and insufficient to support routine pharmacogenomic testing in clinical practice [211,212]. In this context, ML based on multiple sources of information represents a novel approach that may contribute to the identification of robust predictor variables.

In a meta-analysis of 20 studies, Lee et al. showed that ML was able to predict therapeutic outcomes among subjects of previously published interventional studies (pharmacological, neuromodulatory, psychotherapy, or combined interventions) with an overall accuracy of 82% based on four predictor types (neuroimaging, phenomenological, genetic, or combined) [172]. As the authors mentioned, ML should now be tested in prospective trials.

Similarly, Pigoni et al. reported that ML could be a valid approach to identifying predictors of treatment-resistant depression, as well as predictors of response to pharmacological and non-pharmacological treatment in patients with treatment-resistant depression [213]. The most common definition of treatment-resistant depression requires at least two prior failures of pharmacotherapy and confirmation of a prior adequate dose and duration [214].

##### ML in the Early Diagnosis of Psychosis

Schizophrenia is a life-long mental disorder with a 1% lifetime prevalence [215]. The early onset, low remission rate, high disability associated with schizophrenia, and premature mortality due to higher rates of comorbid physical conditions, such as metabolic and cardiovascular diseases, lead to a significant burden of disease (years of life lived with disability and years of life lost to premature mortality) [216]. Therefore, there has been considerable academic and clinical interest in individuals with a high risk of developing schizophrenia.

A set of criteria has been determined to identify young high-risk individuals. The state of clinical high risk requires the fulfilment of at least one of the following criteria: attenuated psychotic symptoms (APS; subthreshold psychotic symptoms), brief limited intermittent psychotic symptoms (BLIP; full-blown psychotic symptoms for a maximum of a week), and genetic risk plus deterioration syndrome (GRDS; family history of schizophrenia or schizotypal personality and marked decline in functioning) [217,218]. High-risk individuals show a considerable risk of transition to schizophrenia, namely, 18% after 6 months of follow-up, 22% after 1 year, 29% after 2 years, and 36% after 3 years [219]. Therefore, identifying high-risk individuals is important. Early detection of a state of clinical high risk and early intervention may help prevent or reverse the transition to psychosis [220].

In a meta-analysis of 11 studies, Fusar-Poli et al. investigated the prognostic accuracy of psychometric instruments in determining the risk of developing a first episode of schizophrenia at a 38-month follow-up in young help-seeking people referred to high-risk services [220]. They found an excellent AUC of 90%, driven by a very high sensitivity of 96%, very much in contrast to the poor specificity of 47%. As the authors mentioned, new strategies are required to increase specificity while preserving sensitivity. ML may contribute to this solution. Sanfelici et al. demonstrated in a meta-analysis that ML applied to clinical and biological data had a sensitivity of 71% and a specificity of 75% [218]. Koutsouleris et al. reported that ML outperformed human prognostication [221]. In the future, ML may significantly contribute to the accurate early detection of a clinical high risk for schizophrenia.

##### Further Areas of Research on ML in Psychiatry

Further promising areas of research regarding ML use in psychiatry include the evaluation of the individual risk for long-term posttraumatic stress disorder (PTSD) based on predictor variables from clinical records, questionnaires, biomedical data, and neuroimaging [222,223,224]. This application of ML could have a significant clinical impact in consideration of emerging evidence for early psychological and pharmacological interventions in individuals at risk for long-term PTSD [225,226].

ML may also prove helpful in the diagnosis of delirium. Delirium is a severe and common neuropsychiatric disorder in hospitalized patients. The incidence in general medical wards is 11–42%, while reaching 87% among critically ill patients [227]. Delirium is associated with double the risk of death and a 13-fold risk of dementia [228]. The diagnosis of delirium is often challenging, and the rate of misdiagnosis ranges from 42% to 64% [229]. In this context, ML may represent an opportunity to improve the diagnostic accuracy in patients presenting with delirium. Hercus and Hudaib reported an AUC of 79%, an accuracy of 72%, a sensitivity of 77%, and a specificity of 67% in a retrospective study that used ML to classify cases of accurate delirium diagnosis versus misdiagnosis [229].

Furthermore, there is emerging evidence for the application of ML in substance-use disorder data, for example, to predict the risk for substance-use disorder or the trajectory of substance-use severity using predictor variables such as demographic data, psychopathology, personality scales, personal and family history, EEG measures, genomic data, and further biomarkers [230,231].

##### Challenges around ML

A key success factor of ML is the quality of the data used for training algorithms [170]. These data need to be specific, multidimensional (e.g., diagnosis, laboratory results, neuroimaging, and genomics), and unbiased; that is, generalizable beyond the training data (e.g., no oversampling of sicker, well-compensated, or healthy populations) [170]. Furthermore, data acquisition must comply with data protection laws and regulations.

ML should be effectively regulated and comply with the quality standards of regulatory agencies. According to Stead, the evidence standard for ML should be proportionate to the task at hand [176]. A higher standard for proof should be required for applications that have a higher clinical impact, for example, diagnosis and treatment [176]. The objective should be to achieve a reasonable balance between innovation, efficacy, and safety [170].

Developers of ML should provide the academic and clinical community with sufficient insight into the intricacies of ML methods to allow for scientific reproducibility and trust building [232,233]. More complex ML models often have greater accuracy but lower interpretability; that is, ease of understanding how the model works [234]. Currently, many algorithms lack transparency [235]. Such “black boxes” may hamper the adoption of ML in clinical practice at the expense of patient care. Brown et al. reported that clinicians were less likely to act on ML-generated suicide risk flags if clinical features built into the algorithm were hidden or not intuitively relevant predictors of suicide risk [236].

Lower interpretability may also adversely affect doctor–patient communication and, hence, the doctor–patient relationship [98]. However, as Hinton pointed out, clinicians, scientists, and regulators should keep in mind that it is generally impossible to capture and interpret all the features used by a deep learning model to reach a conclusion [237]. Liu et al. provided an overview of ML and how to assess the published literature on ML-based tools [238].

Further, the cost-effectiveness of ML in healthcare should be demonstrated, considering cost drivers such as development, maintenance, and updating of algorithms, data storage, data curation, and data visualization [170].

#### 3.2.4. Precision Psychiatry

ML, based on the analysis of big data, opens the door to precision psychiatry. Ultimately, precision psychiatry may lead to the categorization of psychiatric patients into new data-driven subgroups. This novel categorization could improve patient care at various levels: homogenous disease classification, early diagnosis, prediction of disease trajectory, and tailored, more effective, safer, and predictable treatment, potentially at the individual level [173]. This categorization would be based on patterns of biomarkers or endophenotypes such as psychopathology, neuropsychological data (including configured self-reports), neurophysiological data, biochemical data, neuroimaging, electrophysiology, and genetics [158,173,239] (pp. 199–224). These endophenotypes would cut across traditional diagnoses [173].

This approach was supported by the Research Domain Criteria (RDoC) Initiative [240]. This is consistent with the fact that psychopathological symptoms and risk alleles are usually shared among different psychiatric disorders [173]. Therefore, these biologically defined subgroups are unlikely to match the DSM and ICD classifications that describe symptom phenomenology.

ML in psychiatry also requires large amounts of data. Therefore, standardized procedures for data acquisition across clinical centers are needed to collect large amounts of homogenous and comparable data [173]. In the future, data acquisition for ML could be increasingly fueled by real-world data, for example, from EHR, neuroimaging, mental health apps, wearable devices, sensor data, speech, social media feeds, or billing information [60,241,242] (pp. 144–149).

### 3.3. Helping Physicians Manage and Leverage Information

#### 3.3.1. Clinical Decision Support

Clinical decision support (CDS) provides clinicians with knowledge (e.g., treatment guidelines) and patient-specific information (e.g., clinical and laboratory data), specifically selected and presented in a timely fashion, to enhance the quality of medical care [243].

Running in the background of EHR, CDS can provide automated alerts (e.g., in case of abnormal vital signs), automated reminders (e.g., reminding the physician of routine laboratory testing in patients taking psychotropic medications), and information related to drug prescription (e.g., dosage, contraindications, allergies, and interactions) [235,244]. However, information overload that may hinder physicians from carefully evaluating CDS recommendations should be avoided [235].

Furthermore, computerized CDS can provide treatment algorithms, for example, for the evidence-based treatment of depression and schizophrenia [245,246,247,248,249]. However, Bauer et al. pointed out that only a few studies have compared CDS to clinical judgement in decision-making [235]. They also reported that CDS does not seem to outperform the physicians’ clinical judgement. One of the reasons is the difficulty of CDS in handling multimorbidity, in particular, the ability to combine clinical practice guidelines without side effects [250]. This clearly represents a challenge for the use of CDS in psychiatry since approximately one-third of adults with a mental disorder have a co-occurring mental disorder [251].

CDS will benefit from future advances in AI. It is a promising field for digitalization in psychiatry, particularly in routine decision-making [235]. Data quality, the reliability of CDS algorithms (e.g., in terms of risk of programming errors and malfunction due to technical issues), and physicians’ awareness of CDS limitations (e.g., in patients with comorbidities) will be among the key success factors [235]. As Bauer et al. mentioned in their review, CDS should be considered as a strategy to support and enhance rather than replace physicians’ decision-making [235].

#### 3.3.2. EHR

EHR is one of the most valuable sources of big data for ML. However, a few obstacles related to unstructured data need to be overcome before the full potential of EHR can be leveraged [158] (pp. 7–13). First, ML algorithms in one language cannot be applied to other languages. Second, the patients’ verbal expressions are influenced by the sociocultural context. Third, healthcare professionals must ensure the accurate and comprehensive documentation in EHR.

The market for EHR is likely to be transformed by a new competition from tech giants. For instance, Apple created HealthKit, a central repository for health and fitness data on smartphones and smartwatches [252]. Healthcare is one of Apple’s strategic thrusts. As Tim Cook, Apple’s CEO, once put it: “I believe, if you zoom out into the future, and you look back, and you ask the question, ‘What was Apple’s greatest contribution to mankind?’, it will be about health” [253].

#### 3.3.3. Physician Charting

Sinsky et al. reported that physicians in ambulatory care spent only 27% of their total time on direct face-to-face contact with patients, compared with 49% on EHR and deskwork [254]. Similarly, Arndt et al. reported that primary care physicians spent 52% of their workdays on EHR tasks [255]. Coupled with the perceived inefficiencies of EHR, the time-consuming documentation of patient information has fueled the increasing use of scribes; that is, unlicensed individuals hired to enter data into the EHR under clinician supervision [256]. This shows that there is an urgent need to help physicians reduce time spent on EHR tasks and deskwork to allow for increased face time with patients.

This is important for the therapeutic alliance and continuance in care. Rosen et al. demonstrated that the quality of the working alliance and the patient’s continuance in care were significantly lower when the therapist used a computer during the mental health intake session [257]. Focusing on the computer screen, the psychiatrist cannot make eye contact, show empathy, observe the patient’s body language and behavior, and reflect on transference and countertransference [258].

Moreover, it takes too long to create notes and reports entered by keyboard and mouse. A keyboard and mouse also require the use of a fixed workstation, preventing the physician from creating progress notes on a mobile device, for example, during medical rounds; the delay between rounds and availability of notes in the EHR may hamper the treatment process. Additionally, the quality of notes entered by keyboard and mouse may be hampered by the overuse of copy–paste [259].

AI is a part of the solution. Voice dictation can recognize and process the words the physician says, then capture them on a computer or mobile device. It has the potential to reduce the time required for documentation, while maintaining documentation quality, and to enhance the fluidity of the treatment process [259,260].

Some software providers are going one step further by developing digital, artificially intelligent assistants that aim to automate physician charting [60] (p. 107). Running in the background on a mobile device, such as a scribe sitting in the examination room, the application integrates with the EHR and takes on time-consuming tasks such as taking notes where the data need to be in the EHR, generating orders (e.g., medication, diagnostics) and referrals, or scheduling appointments. As with voice dictation, the development and deployment of medical digital assistants is likely to benefit from the rise of AI.

### 3.4. The Doctor–Patient Relationship

#### 3.4.1. Digital Real-Time Language Translators

Artificial intelligence is driving the development of digital real-time language translators. Such digital systems may prove helpful in treating patients from a migrant background, for example, when no suitable interpreter is available. Real-time translation allows for natural conversation flow and saves time. Similar to human interpreters, close attention should be paid to the quality of the translation, since inadequate interpretation can jeopardize the treatment of patients with psychiatric disorders [261,262].

#### 3.4.2. Online Mental Health Resources for Patients

Approximately 20% of the population lives with varying degrees of mental illness [263]. For those who seek medical information on their condition, the internet represents an easily accessible and inexpensive source of information. Powell and Clarke showed that the internet is used as a source of mental health information by approximately 10% of the general population, 15% of those with current psychological distress, and 20% of those with a history of mental health problems [264]. Seeking mental health information on Google was shown to be higher in winter than in summer months across all mental health queries, following the seasonal pattern found for several mood disorders [265]. Yigzaw et al. found that online health information was associated with an increase in physician visits [266]. They suggested that the internet served as a supplement to traditional healthcare services rather than as a replacement.

Health literacy refers to the ability to access, understand, appraise, and apply information relevant to health [267]. According to the WHO, health literacy is a stronger predictor of health status than income, employment status, education level, and racial or ethnic group [267]. Providing meaningful and reliable information is required to improve health literacy [267]. Thus, high-quality online mental health information can contribute to an individual’s mental health.

Health literacy can also facilitate shared decision-making in psychiatry (SDM). SDM can be defined as the process in which the physician provides the patient with clear and complete medical information to help them decide among multiple acceptable treatment options in accordance with his or her preferences [268,269]. In other words, the physician is the expert in the evidence, while the patient is the expert in what matters most to him or her [268]. Online mental health information can help patients acquire relevant medical evidence and develop informed preferences [270]. In a Cochrane review across all conditions, Stacey et al. found high-quality evidence that SDM improves the patients’ knowledge regarding treatment options and reduces the patients’ indecisiveness related to feeling uninformed or unclear about what matters most to them [271].

The implementation of SDM in psychiatry has remained limited, although its use has been widely recommended [272,273]. One reason for this may be the lack of robust empirical evidence [273]. Thus, the authors of a Cochrane review indicated that no firm conclusions can be drawn about the effects of SDM interventions for people with mental health conditions [274]. However, SDM remains a promising strategy in psychiatry, for example, for the treatment of schizophrenia and depression [269,275,276,277].

Nevertheless, seeking health information on the internet may not be beneficial to everyone. Doherty-Torstrick et al. reported that individuals with higher levels of illness anxiety recall experiencing more anxiety during and after searching [278]. Therefore, psychiatrists should recommend such people to avoid symptom-searching on the internet [278].

Online physician rating is another internet resource for patients that exerts an influence on the doctor–patient interaction. Physician-rating websites (PRW) provide insight into the quality of care from the patient’s perspective [279]. Hanauer et al. reported that approximately 60% of people consider PRW important when choosing a physician [280]. However, several data quality issues affecting PRW have been described, such as accuracy, relevance, objectivity, timeliness, and completeness [281]. Furthermore, online physician ratings may not reflect the actual quality of care as measured by accepted metrics of therapy outcomes [282].

As Lee mentioned, PRW can be useful in the medical ecosystem [283]. First, peer opinion can help patients make informed decisions. Second, patients’ feedback can help physicians improve their services. Third, physicians who share patient reviews foster transparency and, hence, a trust-based doctor–patient relationship.

According to Lee, the question is not whether information on patient satisfaction should be made public, but rather, who should do it [283]. Murphy et al. recommended that physicians embrace the change process by helping shape future doctor-rating platforms [284].

#### 3.4.3. Digitalization and the Therapeutic Relationship

There is a significant difference in the diagnosis of psychiatric disorders and other medical conditions. According to the ICD-11, psychiatric disorders are syndromes characterized by clinically significant disturbances in an individual’s cognition, emotional regulation, or behavior that reflect a dysfunction in the psychological, biological, or developmental processes that underlie mental and behavioral functioning [43]. The diagnostic criteria for psychiatric disorders are based on the patient’s own observations, others’ observations (including the examiner’s observations), time criteria, the course of disease, and exclusion criteria [43]. Biomarkers (e.g., laboratory findings, omics data, or radiological features) are not mentioned in Chapter 6 of the ICD-11 on mental, behavioral, or neurodevelopmental disorders. Therefore, the psychiatrist’s ability to take the patient’s medical history and to assess his or her psychopathology remains instrumental in diagnosing psychiatric disorders, although many biomarkers have been described [158] (pp. 7–13). However, the patient will only open up to the psychiatrist if there is a trust-based therapeutic relationship.

The therapeutic relationship plays a key role in psychotherapy outcomes. Factors related to the patient (e.g., resources, personality, and therapy motivation), therapist (e.g., skills and personality), and therapeutic relationship account for approximately 30% of success, compared to 40% for extra-therapeutic change (e.g., social support), 15% for factors that are specific to the treatment method (e.g., the use of schema therapy in the treatment of personality disorders), and 15% for expectancy or placebo effect [285]. Therefore, the therapeutic relationship is estimated to account for as much psychotherapy success as the treatment method. In a meta-analysis, the relationship between therapeutic alliance and psychotherapy outcomes was shown to be comparable between face-to-face and eHealth-based psychotherapy, including phone, internet, videoconferencing, and email [203]. The authors concluded that attention should be paid equally to the therapeutic alliance in face-to-face and eHealth-based psychotherapy.

Moreover, the quality of the therapeutic relationship is instrumental in medication adherence in psychiatry [286,287,288].

Therefore, the ability of interpersonally skilled therapists to forge a strong therapeutic alliance with their patients is instrumental to the success of psychotherapy and pharmacotherapy.

Sucala et al. reported that providing digital mental health services through technologies such as video conferencing, chat technology, or email seemed to be at least equivalent to face-to-face therapy in terms of therapeutic alliance, and that there was a relationship between the therapeutic alliance and the outcome of digital mental health interventions [289].

## 4. Conclusions

The digital revolution is innovating healthcare as it is many other industries. The pace of digital innovation, the need for social distancing, and the shortage of medical resources in the context of the COVID-19 pandemic, as well as considerable demographic strain on the healthcare system, catalyze the development and implementation of innovative technologies and digital services in healthcare. Furthermore, the use of technology is common in the Digital Native (Z), Millennial (Y), and X Generations [290]. Psychiatry has no other choice but to embrace the change.

Various eHealth services, such as telepsychiatry, C-CBT, I-CBT, and app-based interventions represent effective, scalable, and cost-efficient options for providing treatment to people with limited or no access to mental health care. Therefore, these eHealth services are synergistic with the resolution of the United Nations on universal health coverage [291].

ML is likely to advance psychiatry by helping design reliable assessments, providing new insights into homogeneous disease classification, allowing early diagnosis of complex psychiatric disorders, predicting disease trajectory, forecasting treatment outcomes, and developing more effective and safer treatments, potentially at the individual level. ML will benefit from the increasing volume of big data (e.g., from omics, neuroimaging, and real-world data) and from a quantum leap in computational sciences. ML is spearheading the advancement of psychiatry toward precision medicine.

VR, applied games, and holographic video conference systems may be the next fast-growing technologies in psychiatry, leading to a stream of innovative psychotherapies. Conversely, the future of the DMS remains unclear. While digital aripiprazole has been approved by the FDA, the application for marketing authorization was withdrawn in Europe, the pharmaceutical company citing the inability to resolve the European Medicines Agency’s concerns [292].

In the future, user-friendly EHR, AI-based CDS, voice dictation, automated physician charting through digital artificially intelligent assistants, and digital real-time language translators may allow psychiatrists to spend more time interacting with their patients and focusing on the face-to-face or online doctor–patient relationship. Even in the digital age, the therapeutic alliance will remain a precondition for successful treatment outcomes in psychiatry. The doctor–patient relationship may also benefit from patient-focused online mental health resources through improved SDM, trust-building physician-rating platforms, and holographic video conference systems through enhanced blending of all communication channels (verbal, vocal, facial, and gestural channels).

There is certainly a risk that physicians might be flooded by the amount of digital information flowing into the patient’s EHR. For this, digitalization may not only be a curse, but also a blessing. Automated CDS always running in the background may help physicians detect risks early, make evidence-based decisions, and manage the complexity of continuous data streams [241].

Digital technologies that have a clinical impact (e.g., ML) and eHealth services should be effectively regulated and comply with high standards for efficacy, safety, and privacy. At the same time, a reasonable balance needs to be reached between these standards and innovation so that patients can benefit from the full potential of new digital technologies. An example of a regulatory model is provided by the FDA’s Digital Health Software Precertification (Pre-Cert) Program [293].

Staying current with new technologies is a significant challenge for physicians. Faculty, trainees, and clinicians need new knowledge and skills to ensure quality care [294]. This requires a new approach to teaching and clinical supervision [294]. Medical professional societies play an important role in providing specific continuing education and access to guidelines on digital tools and services.

Industry, faculty, clinicians, and other stakeholders should agree on data standards that allow for sharing, exchanging, and combining data, duly taking data privacy into account. Data standards are a prerequisite for leveraging the full potential of big data in AI-based research.

In our hospital, as in many other psychiatric clinics worldwide, the COVID-19 pandemic served as an enhancer for digitalization [295]. Digitalization has become a strategic thrust in psychiatry. The integration of digital and traditional healthcare into “digital clinics” will be instrumental in harnessing the full potential of new digital technologies.

Psychiatry in the digital age: A blessing or a curse? The objective of digitalization in psychiatry is not to replace traditional psychiatric care through digital therapies, but to augment it through innovative interventions [296]. To use Tim Cook’s words, if you zoom out into the future, look back, and ask the question, “What was digitalization’s greatest contribution to psychiatry?”, it will be about the therapeutic alliance. We believe it is a blessing.

## Data Availability

Not applicable.
